# Efficacy of SAT2 Foot-and-Mouth Disease Vaccines Formulated with Montanide ISA 206B and Quil-A Saponin Adjuvants

**DOI:** 10.3390/vaccines9090996

**Published:** 2021-09-07

**Authors:** Ntungufhadzeni M. Rathogwa, Katherine A. Scott, Pamela Opperman, Jacques Theron, Francois F. Maree

**Affiliations:** 1 Vaccines and Diagnostic Development, Onderstepoort Veterinary Research, Agricultural Research Council, Onderstepoort 0110, South Africa; mactungu@gmail.com (N.M.R.); katherineanne.scott@astrazeneca.com (K.A.S.); StoreyP@arc.agric.za (P.O.); 2 Department of Biochemistry, Genetics and Microbiology, Faculty of Natural and Agricultural Sciences, University of Pretoria, Pretoria 0002, South Africa; jacques.theron@up.ac.za; 3 Department of Production Animal Studies, Faculty of Veterinary Science, University of Pretoria, Pretoria 0110, South Africa

**Keywords:** foot-and-mouth disease virus, stabilized antigen, adjuvant, vaccine, SAT2

## Abstract

The effective control of foot-and-mouth disease (FMD) relies strongly on the separation of susceptible and infected livestock or susceptible livestock and persistently infected wildlife, vaccination, and veterinary sanitary measures. Vaccines affording protection against multiple serotypes for longer than six months and that are less reliant on the cold chain during handling are urgently needed for the effective control of FMD in endemic regions. Although much effort has been devoted to improving the immune responses elicited through the use of modern adjuvants, their efficacy is dependent on the formulation recipe, target species and administration route. Here we compared and evaluated the efficacy of two adjuvant formulations in combination with a structurally stabilized SAT2 vaccine antigen, designed to have improved thermostability, antigen shelf-life and longevity of antibody response. Protection mediated by the Montanide ISA 206B-adjuvanted or Quil-A Saponin-adjuvanted SAT2 vaccines were comparable. The Montanide ISA 206B-adjuvanted vaccine elicited a higher SAT2 neutralizing antibody response and three times higher levels of systemic IFN-γ responses at 14- and 28-days post-vaccination (dpv) were observed compared to the Quil-A Saponin-adjuvanted vaccine group. Interestingly, serum antibodies from the immunized animals reacted similarly to the parental vaccine virus and viruses containing mutations in the VP2 protein that simulate antigenic drift in nature.

## 1. Introduction

Foot-and-mouth disease (FMD) is a highly infectious, vesicular disease that affects cloven hoofed animals and remains a major threat to livestock production and livestock-derived industries worldwide. In southern Africa, the control of FMD relies strongly on the separation of wildlife from susceptible livestock and vaccination of cattle in high risk areas neighboring national parks. The Office International des Epizooties (OIE) advocates a zoning system for the control of FMD where an FMD-free region is separated from an infected area (endemic area, game parks) by a zone where vaccination of cattle or other susceptible livestock and continuous surveillance are practiced [1]. Accordingly, in South Africa, FMD control depends on tri-annual vaccination of susceptible cattle in the high risk area, surrounding the Kruger National Park (KNP) and adjacent game farms, with a prophylactic vaccine containing antigen from all three Southern African Territories (SAT) serotypes [2,3]; fences separating animals at the wildlife-livestock interface [4,5]; movement restriction between zones and continued surveillance to allow early FMD detection [5].

Control of FMD by means of vaccination is based on the chemical inactivation of complete viral 146S particles [6,7]. Despite the successful use of FMD vaccines in North America and Europe [8], the effective administration of vaccines in developing countries is hampered by several factors including lack of inducing a long-lasting protective immune response; low thermal stability of the vaccine antigen [7]; the use of two dose regimes administered 4–6 weeks apart and the inability to cross-protect against multiple genetic and antigenic lineages within a serotype [3,9]. Consequently, frequent booster vaccinations are required, which are expensive, time-consuming, reliant on cold chain and require human resources to administer. Moreover, the structural integrity of the intact 146S antigen contained in the vaccine may influence the induction of a protective immune response [10,11,12]. Therefore, more stable vaccines are hypothesized to improve the duration of protective immune responses in animals. The use of a reverse genetic approach and targeted mutagenesis [13] has provided a valuable tool for genetic manipulation of the virus to improve viral properties such as cell culture adaptation, biophysical stability, and broadened cross-reactivity [11,12,14,15,16].

Many efforts are devoted to improving immune responses through the use of modern adjuvants [17,18,19]. Although a variety of adjuvants such as oil emulsions, saponin, and mineral salts for veterinary use are widely available, their efficacy is dependent on the formulation recipe, target species, and administration route [3]. The selection of a suitable adjuvant is an important factor in determining the efficacy of FMD vaccines. The adjuvants most frequently used for inactivated FMD vaccines are aluminum hydroxide (Al(OH)_3_; AL) and mineral oil-based adjuvants with or without saponin [3,20,21].

Vaccines containing AL and crude saponin as adjuvants have several deficiencies such as toxicity, causing haemolysis at the injection site and inducing short-lived antibody responses [18,20,22,23]. Quil-A adjuvant, a water-extractable fraction of saponin, is less toxic, safe to use, superior in inducing cell-mediated and antibody-mediated immune responses than crude saponin and is simple to formulate [24]. The use of oil adjuvanted vaccines in cattle can improve the duration of immunity by at least six months, thus reducing the frequency of vaccination [3,18,25,26,27]. Although oil adjuvanted vaccines elicit a superior immune response, it can cause side-effects such as a localized reaction (lesions) and swelling at the injection site [18,19,20,22]. Nevertheless, the advantages of Quil-A and oil adjuvanted FMD vaccines outweigh that of conventional adjuvants, because the vaccine retains its potency for a longer period following storage at 4 °C and elicits good immune responses regardless of the injection route [18,28]. However, most adjuvant studies conducted in the past were either based on crude saponin, which is toxic, or the experiments were performed on species other than cattle and using FMDV antigen other than SAT serotypes [22,26].

In this study, we compared and evaluated the efficacy of two adjuvant formulations in combination with a structurally stabilized SAT2 vaccine antigen, designed to have improved thermostability [11,12]. The ability of FMD vaccines adjuvanted with either a double oil emulsion (W/O/W) of Montanide ISA 206B or a water-based Saponin-extract, Quil-A, to elicit protective immune responses in Nguni cattle was investigated. The immune responses generated in cattle were assessed using total and neutralizing antibodies, IgG isotyping and gamma-interferon (IFN-γ) responses to study T-lymphocyte regulated cell-mediated immunity against the FMD vaccine antigen.

## 2. Materials and Methods

### 2.1. Cells and Viruses

Baby hamster kidney (BHK) cells, strain 21, clone 13 (ATCC CCL-10), were propagated as described previously [29] and used for vaccine antigen production. Instituto Biológico renal suino-2 cells (IB-RS-2) were maintained in Roswell Park Memorial Institute (RPMI) medium (Sigma-Aldrich, Merck, Germany) supplemented with 10% fetal calf serum (FCS) (Delta Bioproducts) and used for virus isolations, titrations by serial dilution and as the indicator cells in the virus neutralization test (VNT) [30]. A structurally stabilized vSAT2-S93H mutant virus derived from the SAT2/ZIM/7/83 virus, described previously [11,12], was used for the production of binary ethyleneimine (BEI)-inactivated FMD vaccine antigen (146S particles). The epitope-modified viruses vKNP^S2a^SAT2 and vKNP^S2b^SAT2 [31] were used to quantify the relative amount of total antibodies that react against epitopes known to change during the emergence of a drifted FMDV strain. The vKNP^S2a^SAT2 and vKNP^S2b^SAT2 viruses contain amino acid changes constructed in the SAT2/ZIM/7/83 genome-length clone, vSAT2, and represent a variation observed at two antigenic sites of SAT2 viruses involving residues 71 to 72 and 133 to 134 of the VP2 protein.

### 2.2. Production of FMDV Antigen and Vaccine Formulation

Supernatant of cultured BHK-21 cell monolayers infected with vSAT2-S93H were harvested, clarified by centrifugation and inactivated with 5 mM BEI (0.875 N NaOH and 0.5 mM BEA (Sigma-Aldrich) in water, pH 7.4) for 26 h at 28 °C [32]. The inactivated antigen was clarified by centrifugation for 30 min at 8000 rpm, concentrated with 8% (*w*/*v*) polyethylene glycol (PEG-8000) (Sigma-Aldrich) for 3 h at 4 °C and purified on a 10–50% (*w*/*v*) sucrose density gradient (SDG) [33], by rate zonal centrifugation at 16,000 rpm for 17 h at 4 °C. The gradients were fractionated and peak fractions containing 146S virion particles (extinction coefficient E_259nm_ = 79.9 [34]) were pooled for vaccine formulation. The integrity of antigen viral RNA was verified by RT-PCR [16] and sequencing of the P1/2A-coding region. The vKNP^S2a^SAT2 and vKNP^S2b^SAT2 viruses were purified similarly for use in a competition ELISA.

The purified 146S particles were subjected to a sterility assay for quality control purposes as described in [9]. Two separate vaccine formulations, incorporating inactivated 146S vSAT2-S93H antigen with commercial semi-purified water-based (water-extractable fraction) Quil-A Saponin (InVivoGen, San Diego, CA, USA) and double oil emulsion Montanide ISA 206B (Seppic, Courbevoie, France), were prepared. The antigen was diluted in Tris/KCl buffer (0.1 M Tris, 0.3 M KCl, pH 7.5) for each vaccine to contain 6–8 µg of antigen per 2 mL dose, which is within the 2–10 µg per 2 mL dose range of standard FMD vaccines. Subsequently, adjuvants were mixed in the aqueous antigen phase in a 1:1 ratio (*v*/*v*) at 30 °C for 30 min, rendering an oil-in-water and a water-in-oil-in-water emulsion, respectively, and stored at 4 °C for 24 h. This was followed by a second brief mixing cycle for 5 min. Both vaccines were stable for at least six months stored at 4 °C. A 1× PBS-only placebo vaccine was also included.

### 2.3. Cattle Immunizations and Virus Challenge

Sixteen naïve Nguni cattle (6–9 months of age and weighing 150 kg) sourced from an FMD-free area were divided randomly into two groups, each consisting of seven animals (*n* = 7) and a third control group containing two animals (*n* = 2). All procedures were approved by the Agricultural Research Council (ARC), Onderstepoort Veterinary Research (OVR) Animal Ethics Committee (AEC) (AEC 25.12) and the Department of Agriculture, Forestry and Fisheries (DAFF) Section 20 permit (10/04/2013). The absence of antibodies to FMDV was confirmed with ELISA (see below) prior to immunization. After an acclimatization period, the cattle were vaccinated intramuscularly on days 0 and 42 with a 2-mL vaccine dose (6–8 µg of vSAT2-S93H antigen). The control animals received a placebo vaccine containing PBS only. Clotted and heparinized blood was collected 6 days prior to vaccination, every 2 days from day 0 to 14 and 21 post-vaccination (pv), thereafter every fortnight until 162 days post-vaccination (dpv). Cattle were kept and allowed to roam freely in a 0.3 hectare camp at ARC-OVR, Kaalplaas, Onderstepoort.

At 150 dpv, cattle were brought into the biosafety level 3 (BSL-3) high-containment animal facility at Transboundary Animal Disease (TAD) of the ARC-OVR. Each group of cattle was housed separately and acclimatized for 12 days. On 162 dpv, immunized and control groups were sedated and inoculated intra-dermolingually at two sites, each with 1 mL of 10^4^ TCID_50_ cattle-adapted homologous SAT2/ZIM/7/83 challenge FMD virus. Clotted and heparinized blood was collected on days 0, 2, 4, 7, 9, 11, and 14 post-challenge (pc), along with oropharyngeal (OP) fluid (probang) and retropharyngeal tonsil swabs. The animals were examined daily for fever (mild = 39.5 °C and severe ≥40 °C) and clinical signs in the mouth, tongue, and feet (small lesion/healing vesicles = 1; moderate vesicles = 2; and severe lesion = 3). Animals were euthanized at 14 days post-challenge (dpc) and the carcasses incinerated.

### 2.4. Antibody Detection in Nguni Cattle

Total antibody titres in vaccinated cattle were detected with a SAT/ZIM/7/83-specific liquid-phase blocking enzyme-linked immunosorbent assay (ELISA) (LPBE) [15,35]. Antibodies were measured in serum collected on days 0, 2, 4, 7, 9, 11, 14, and 21 pv and thereafter every fortnight until 162 dpv. Serum samples from 0–11 dpc were also tested.

The reactivity of antibodies in 162 dpv sera were also tested in triplicate wells against the vKNP^S2a^SAT2 and vKNP^S2b^SAT2 viruses in a competition ELISA format, as described previously by Opperman et al. (2014) [31]. Briefly, flat-bottom 96-well plates were coated with rabbit SAT2 antiserum in 50 mM carbonate buffer (pH 9.6). SDG-purified (60 ng/well) vKNP^S2a^SAT2, vKNP^S2b^SAT2 and parental viruses were applied. Sera (162 dpv) was diluted (1:6) in blocking buffer and antibodies detected via competition with monoclonal antibody GD12 (1:40) and horseradish peroxidase (HRP)-conjugated rabbit anti-mouse IgG (Sigma-Aldrich) (1:7000). Following color development, the reaction was stopped and the (OD_450_) values measured. The maximum OD_450_ value was calculated from the negative control and the average readings of two ELISAs were used.

### 2.5. Non-Structural Protein (NSP) ELISA

Serum collected 6 days prior to immunization, 0–162 dpv and 0–11 dpc were tested for NSP antibodies. The PrioCHECK^®^ FMDV NSP Antibody ELISA kit (Thermo Fisher Scientific, Waltham, MA, USA) [36] was used according to the manufacturer’s instructions. Serum with percentage inhibition (PI) values of ≥50% was regarded as positive (indicating the presence of antibodies against the 3ABC protein of FMDV) and a PI value <50% as negative.

### 2.6. Virus Neutralization Test (VNT)

Neutralizing antibodies to SAT2/ZIM/7/83 in serum samples collected at 0, 7, 21, and 28 dpv, thereafter fortnightly until 162 dpv and 2, 4, and 9 dpc were measured with a VNT [9] using IB-RS-2 cells as the indicator system. The test serum was diluted two-fold, starting with a 1:16 dilution, and mixed with a virus suspension containing approximately 100 TCID_50_ per well. After 1 h of incubation at 37 °C, 3 × 10^5^ IB-RS-2 cells were added to each well. Cell-only controls were included in each plate, and a virus titration control and positive serum control were also performed. After 72 h at 37 °C in a humid atmosphere containing 5% CO_2_, the plates were analyzed microscopically and colorimetrically for cytopathic effect (CPE). The 50% end-point serum titres were calculated according to the method of Karber (1931) [37]. The antibody titres were calculated as log_10_ of the reciprocal of the final serum dilution that neutralized 100 TCID_50_ of virus in 50% of the wells.

### 2.7. IgG1 and IgG2 Isotyping ELISA

The IgG1 and IgG2 isotype ELISAs were performed according to Capozzo et al. (1997) [38], except that 100 ng of SDG-purified 146S SAT2/ZIM/7/83 was used per well [35]. A series of two-fold dilutions of test sera starting from 1:50 was prepared. Antibodies were detected using HRP-conjugated sheep anti-bovine IgG1 or IgG2 (BD-Serotec, Oxford, UK) at a dilution of 1:750 and 1:1500, respectively. Titres were expressed as the inverse dilution reaching the cut-off value (0.2), calculated as mean OD + 2SD achieved by the FMDV-negative Nguni bovine serum samples (*n* = 23).

### 2.8. Whole Blood Re-Stimulation and Bovine Interferon Gamma (IFN-γ) ELISA

Whole blood assays [39] were performed using 1.5-mL aliquots of heparinized blood, collected from animals at 14, 28, 56, 84 dpv, 2 and 9 dpc and incubated in 24-well cell culture plates (Nunc). For each animal, duplicate wells were stimulated with 10 µg/mL Pokeweed mitogen (PWM) (Sigma-Aldrich, Merck, Germany) as a positive control stimulator of all IFN-γ, 10 µg/mL purified inactivated 146S SAT2/ZIM/7/83 particles or 1 × PBS as a negative control. Plates were incubated at 37 °C with 5% CO_2_ for 48 h and plasma collected. The Bovine IFN-γ specific ELISA Assay Kit (BD-Serotec, Oxford, UK) used for this assay contains two different mouse anti-bovine IFN-γ monoclonal antibodies and recombinant bovine IFN-γ as a standard (0.025–50 ng/mL) [40]. The levels of stimulated IFN-γ were expressed in ng/mL of plasma.

### 2.9. Virus Isolation

FMDV in oropharyngeal (OP) fluid and retropharyngeal tonsil swabs was detected by inoculation on IB-RS-2 cells as described by [9]. The supernatant was blind passaged at least twice or until CPE was observed. A SAT2-specific antigen ELISA [41,42] was used to confirm the presence of SAT2 virus in cultures showing cytopathic effect (CPE).

### 2.10. RNA Extraction, cDNA Synthesis and Real-Time Quantitative RT-PCR

The FMD viral RNA in OP fluid, retropharyngeal tonsil swabs and heparinized blood was detected with a one-step real-time RT-PCR assay targeting the 3D region [43]. Total RNA was extracted with the QIAamp Viral RNA kit (Qiagen, Hilden, Germany) according to the manufacturer’s specifications. The real-time RT-PCR was carried out in duplicate using a SuperScript III One-step RT-PCR system kit with Platinum Taq DNA polymerase (InVitrogen, Waltham, MA, USA) [43]. Positive test and control samples had Ct values <30, samples with Ct values of 30–39.9 were designated as weak positive, whilst samples with Ct values ≥40 were considered negative.

### 2.11. Statistical Analysis

Antibody titres obtained by LPBE, IgG-isotype ELISAs and in vitro VNT, respectively, were compared by ANOVA 2-factor repeated measures followed by Bonferroni’s multiple comparisons test. The Mann-Whitney test was used when data from two groups were compared. All statistical analyses were performed using GraphPad Prism Software v7.03 for Windows (GraphPad Prism Software, Inc., San Diego, CA, USA) and the confidence interval (CI) was 95%.

## 3. Results

A vaccine challenge study was designed to compare the efficacy of a vaccine consisting of a structurally stabilized SAT2 146S antigen formulated with either a Montanide ISA 206B or a Quil-A Saponin adjuvant, in Nguni cattle. The vSAT2-S93H mutant virus, its growth characteristics, plaque morphologies, ability to grow to high titres in BHK-21 cells and improved biophysical stability have been described previously [11,12].

### 3.1. Antibody Kinetics in Vaccinated Nguni Cattle with Stabilized SAT2 Antigen Formulated with Montanide ISA 206B or Quil-A Saponin Adjuvants

**Figure 1:**
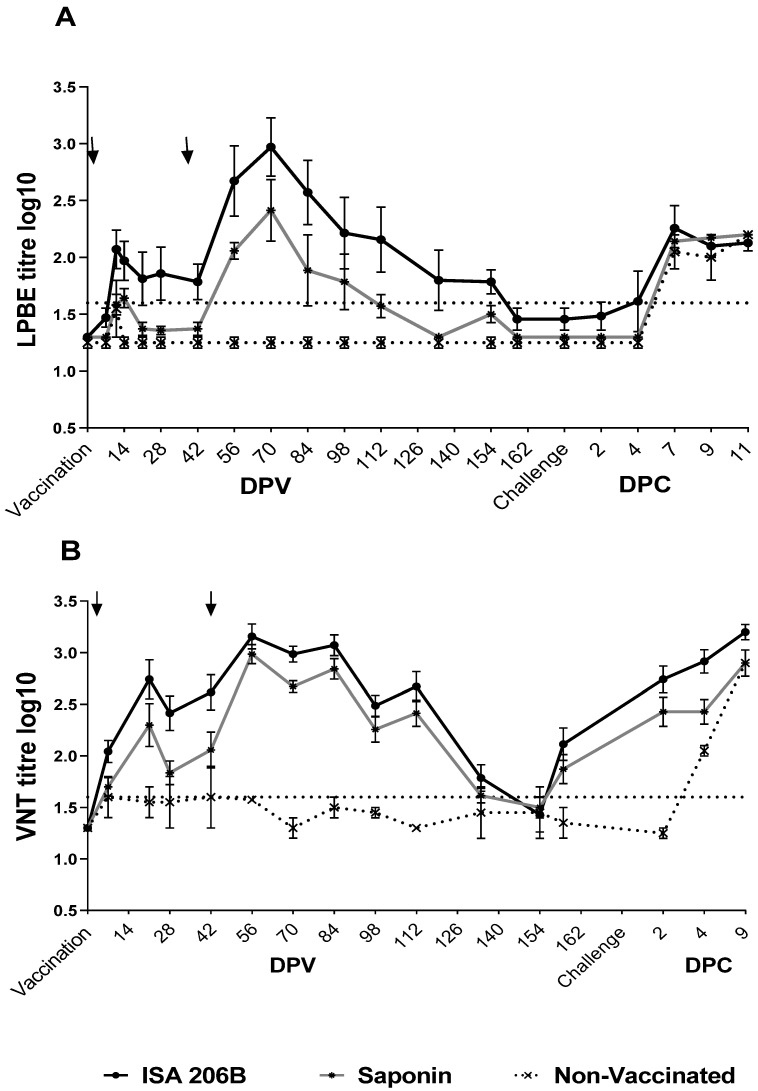
Antibody kinetics in Nguni cattle immunized twice with 2 mL of 6–8 µg dose of vSAT2-S93H BEI-inactivated, SDG-purified antigens formulated with either Montanide ISA 206B (*n* = 7) or Quil-A Saponin adjuvant (*n* = 7) and challenged with SAT2/ZIM/7/83. (**A**) Mean antibody titres (log_10_) were measured by LPBE and (**B**) neutralizing antibody titres (log_10_) were measured by VNTs. Animals regarded as negative had log_10_ titres ≤ 1.6, as shown by the non-vaccinated control animals (*n* = 2). Arrows indicate the time of primary (0 dpv) or secondary vaccination (42 dpv). The dotted line indicates the positive cut-off value for the LPBE or VNT. Error bars represent the standard deviation.

The vaccine efficacy of the structurally modified SAT2 antigen formulated with the Montanide ISA 206B or the Quil-A Saponin adjuvants was determined by the antibody response elicited in Nguni cattle. Serum collected from 0 to 162 dpv was used to measure antibodies against structural proteins (LPBE) and NSP. The absence of NSP antibodies confirmed the absence of FMDV exposure in cattle prior to immunization (baseline) until 162 dpv. Following immunization, the total SAT2 antibody titres showed that cattle vaccinated with either the SAT2-Montanide ISA 206B or the Quil-A Saponin vaccine seroconverted between 9 and 14 dpv and anti-SAT2 antibodies peaked at 14 dpv following primary vaccination (Figure 1A). However, animals vaccinated with the SAT2-Quil-A Saponin vaccine had substantially lower antibody titres (1.7 ± 0.2 log_10_) that dropped below the positive cut-off level (<1.7 log_10_) from 21 dpv compared to the higher titres of the SAT2-Montanide ISA 206B vaccinated group (2.0 ± 0.3 log_10_). After receiving a second vaccination at 42 dpv, there were significant differences (*p* < 0.01) in the total antibody titres between the vaccinated groups, with average titres of 2.7 ± 0.7 log_10_ and 2.1 ± 0.2 log_10_ at 56 dpv for the groups receiving the Montanide ISA 206B and Quil-A Saponin adjuvanted vaccines, respectively. At 70 dpv, four weeks after the second vaccination, the SAT2-Montanide ISA 206B and SAT2-Quil-A Saponin groups attained the highest levels of anti-SAT2 antibody titres with average titres of 3.0 ± 0.4 log_10_ and 2.4 ± 0.6 log_10_, respectively. The antibody titres decreased to 1.4 ± 0.2 log_10_ and 1.3 ± 0.1 log_10_, respectively, by 162 dpv.

Challenge enhanced the antibody response in vaccinated animals with titres increasing between 4 (1.7 log_10_) and 7 dpc (2.2 log_10_) in animals receiving the SAT2-Montanide ISA 206B vaccine, whilst the SAT2-Quil-A Saponin (2.1 log_10_) and control groups (2.0 log_10_) titres increased from 7 dpc. Both vaccinated groups had high titres of SAT2 antibodies (>2.2 log_10_) as animals cleared the circulating virus between 7 and 11 dpc.

Similar antibody kinetic profiles were observed using the LPBE and VNT (Figure 1B), but VNT displayed higher sensitivity with neutralizing antibody titres of 2.1 ± 0.3 log_10_ and 1.9 ± 0.3 log_10_ still present at 162 dpv for the SAT2-Montanide ISA 206B and SAT2-Quil-A Saponin groups, respectively. Significantly (*p* < 0.05) higher anti-SAT2/ZIM/7/83 neutralizing antibody titres were elicited in animals that received the SAT2-Montanide ISA 206B vaccine (2.4 ± 0.3 log_10_) compared to the SAT2-Quil-A Saponin (2.0 ± 0.3 log_10_) vaccinated group at 7–42 dpv following primary vaccination (Figure 1B). Following the second vaccination at 42 dpv, neutralizing antibodies titres peaked at 56 dpv for both groups, with an average of 3.2 log_10_ for the SAT2-Montanide ISA 206B group and 2.9 log_10_ for the SAT2-Quil-A Saponin group. No significant difference (*p* > 0.05) in neutralizing antibody titres was observed between the vaccinated groups at 56 dpv, as well as from 84 to 162 dpv prior to challenge.

The mean neutralizing antibody titres for the animals fully protected by the Montanide ISA 206B and Quil-A Saponin formulated vaccines were 2.7 (1.2–3.5) and 2.2 (1.2–3.0) log_10_, respectively. The unprotected animal that developed systemic FMD in the Quil-A Saponin group had a positive neutralizing antibody titre (2.1 log_10_), which was similar to the protected animal titres.

### 3.2. IgG Isotyping and IFN-γ Responses

**Figure 2:**
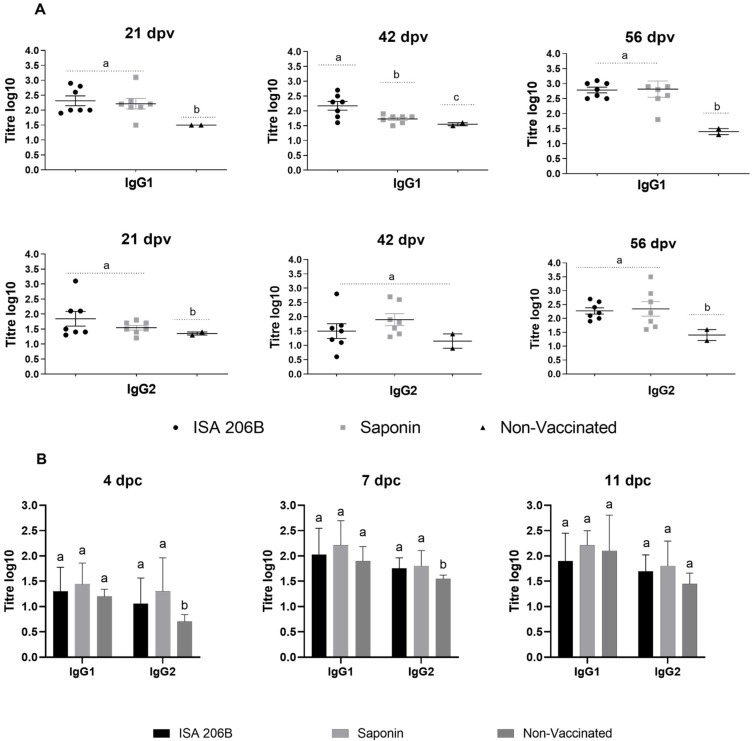
(**A**) Kinetics of anti-FMDV IgG isotype (IgG1 and IgG2) antibody titres at 21, 42, and 56 dpv of Nguni cattle immunized twice with either the SAT2-Montanide ISA 206B or SAT2-Quil-A Saponin vaccines. (**B**) The mean IgG1 and IgG2 titres (log_10_) of cattle, 0–11 days after challenge with SAT2/ZIM/7/83. Error bars represent the standard deviation. Statistical significance is indicated by a, b, or c.

The induction of high levels of serum FMDV-specific IgG1 isotype antibodies has been correlated to protection in vaccinated cattle [38]. The IgG1 and IgG2 anti-FMDV titres in the serum of vaccinated animals were measured at 21, 42, 56 dpv and 4, 7, 11 dpc (Figure 2), and the ratio of IgG1:IgG2 was calculated. Both vaccinated groups induced higher titres of anti-FMDV IgG1 isotype antibodies compared to IgG2 antibody responses. Statistical differences (*p* > 0.05) between titres in the two vaccinated groups were observed (Figure 2A); the mean IgG1:IgG2 ratio for SAT2-Montanide ISA 206B vaccinated cattle were higher (1.5:1, 1.5:1) compared to SAT2-Quil-A Saponin vaccinated cattle (1.3:1, 0.8:1) at 21 and 42 dpv. No discernible difference in the IgG1:IgG2 ratio for both groups was observed at 56 dpv (0.8:1). After challenge the kinetics of IgG isotype titres increased from 4 to 7 dpc in vaccinated animals with IgG1 titres higher compared to the IgG2 titres (Figure 2B). The overall mean IgG1 antibody titres for the animals fully protected by SAT2-Montanide ISA 206B (*n* = 7) was 2.3, 2.2 and 2.8 log_10_ and for SAT2-Quil-A Saponin (*n* = 6) vaccine was 2.0, 1.7 and 2.8 log_10_ at 21, 42 and 56 dpv, respectively.

**Figure 3:**
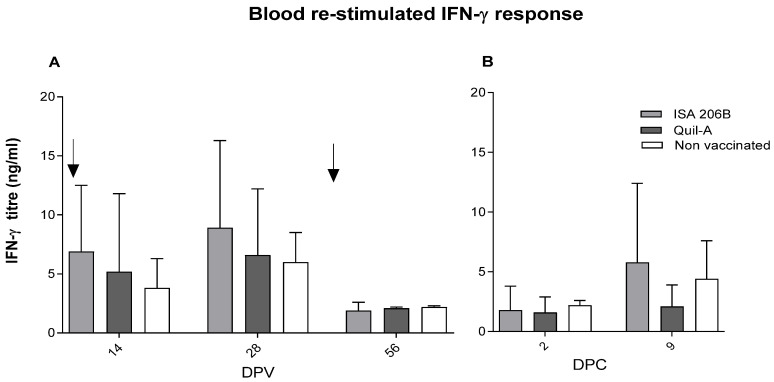
Re-stimulated cell-mediated interferon gamma (IFN-γ) responses. Mean serum titres of IFN-γ (ng/mL) of Nguni cattle after being vaccinated twice with vSAT2-S93H antigen formulated with Montanide ISA 206B (*n* = 7) or Quil-A Saponin adjuvant *(n* = 7) and non-vaccinated (*n* = 2) animals (**A**) at 14, 28, 56 dpv and (**B**) 2 and 9 dpc. Error bars represent the standard deviation. Arrows indicate the time of primary (0 dpv) and secondary vaccination (42 dpv).

Higher titres of systemic IFN-γ were detected at 14 dpv, which peaked at 28 dpv with SAT2-Montanide ISA 206B (8.9 ± 7.4 ng/mL), followed by SAT2-Quil-A Saponin (6.6 ± 5.6 ng/mL) and placebo-vaccinated (6.0 ± 2.5 ng/mL) animals (Figure 3). After challenge, the levels of IFN-γ induced by the SAT2-Montanide ISA 206B group at 9 dpc were significantly (*p* < 0.05) higher (5.8 ± 6.6 ng/mL) when compared with the SAT2-Quil-A Saponin group (2.1 ± 1.8 ng/mL) and placebo-vaccinated animals (4.4 ± 3.2 ng/mL) (Figure 3).

### 3.3. Protection of Vaccinated Cattle against Live SAT2/ZIM/7/83 Virus Challenge

**Figure 4:**
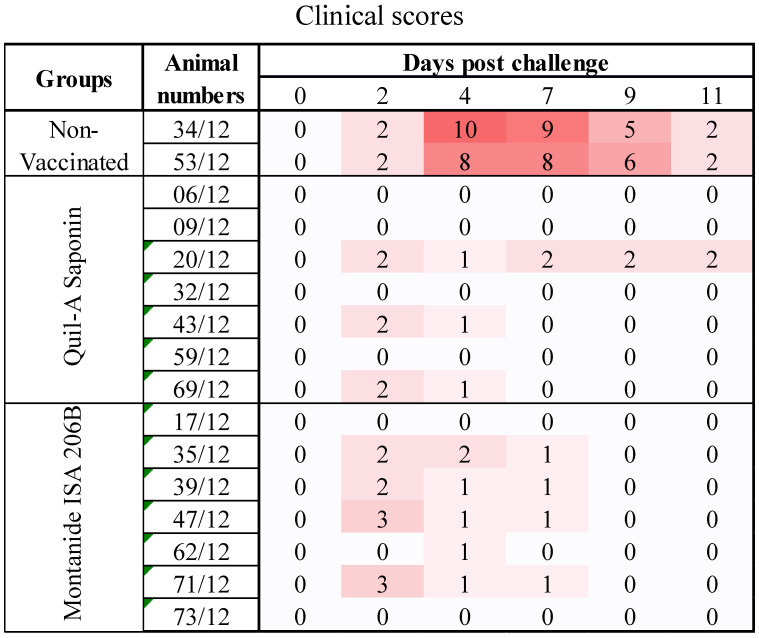
Heat-map depicting clinical scores of Nguni cattle vaccinated with vSAT2-S93H formulated with Montanide ISA 206B (*n* = 7) or Quil-A Saponin (*n* = 7) adjuvant and non-vaccinated animals (*n* = 2), challenged at 162 dpv with SAT2/ZIM/7/83 virus. Cattle were inoculated intra-dermolingually at two sites each with 1 mL of 10^4^ TCID_50_ of cattle-adapted SAT2/ZIM/7/83 virus. The clinical scores were calculated as described in Materials and Methods, with 0–12 indicating low to high severity.

At 162 dpv, cattle were challenged and monitored daily for clinical signs (Figure 4). The SAT2-Montanide ISA 206B vaccinated group (*n* = *7*) was fully protected (100%) against systemic spread of FMD as observed by the absence of generalized lesions on their hooves, whilst mild fever (≥39 or <40 °C) was present in three of the animals from 1 to 4 dpc. One animal in this group had severe fever (40 °C) within 24 h of challenge but subsided at 2 dpc. Of the seven cattle that received SAT2-Quil-A Saponin vaccine, six animals were protected (85.7%). The one unprotected animal had mild (≥39.2 or <40 °C) fever at 2 to 4 dpc and developed moderate clinical vesicular lesions (highest clinical score = 2) on the hind feet 7 to 11 dpc. Furthermore, two other animals in this group had mild fever at 2 dpc. This was in contrast to the two placebo-vaccinated control animals that developed mild (≥39.2 or <40 °C) to severe (≥40 °C) pyrexia and severe lesions (highest clinical score = 8) that were generalized to all four hooves within 4 to 7 dpc, in addition to inappetence and lameness.

### 3.4. Virus Isolation and the Presence of Viral RNA Post Challenge

**Figure 5:**
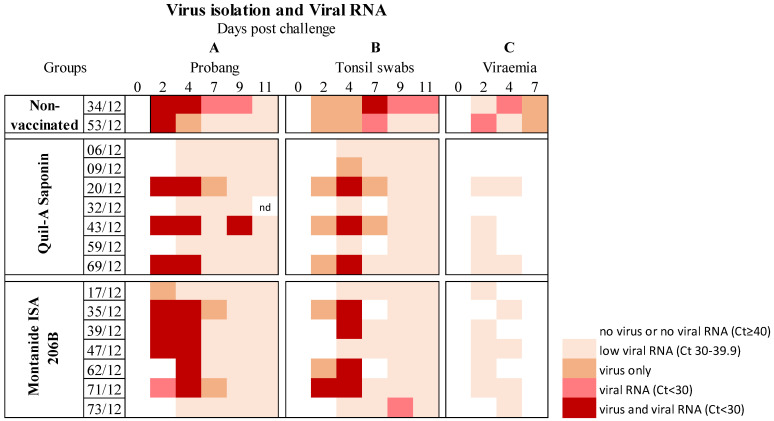
The presence of FMD virus and viral RNA from 0 to 11 days post-challenge displayed on a heatmap. Summary of the detected viral RNA (determined by real-time quantitative RT-PCR) and the presence of virus (recovered by inoculation on IB-RS–2 cells) from (**A**) oropharyngeal (OP) fluid (probang), (**B**) retropharyngeal tonsil swabs and (**C**) heparinized blood samples.

Following challenge, OP fluids, retropharyngeal tonsil swabs, and heparinized blood were collected and subjected to virus isolation on IB-RS-2 monolayer cells. As expected, no virus was isolated from any of the samples on the day of challenge. FMDV was recovered from OP fluid and retropharyngeal tonsil swabs at 2 to 11 dpc (Figure 5). Of the seven animals that received the SAT2-Quil-A Saponin vaccine, at least two animals (20/12 and 43/12) were positive on virus isolation up to 7 dpc and one animal (43/12) for 9 dpc. The SAT2-Montanide ISA 206B vaccinated group showed two animals (35/12 and 71/12) that were positive on virus isolation from OP fluid up to 7 dpc. Generally, virus was recovered from retropharyngeal tonsil swabs at 4 dpc or earlier, two days after OP fluid samples were positive. The SAT2 serotype of FMDV was confirmed by a type-specific antigen ELISA.

FMD viral RNA was detected by quantitative real-time RT-PCR (positive sample Ct value < 30) in OP fluid, retropharyngeal tonsil swabs and from heparinized blood (Figure 5). Three animals from the SAT2-Quil-A Saponin and five animals from the SAT2-Montanide ISA 206B vaccinated groups had viral RNA (Ct < 30) detected from OP fluid and retropharyngeal tonsil swabs at 2 and 4 dpc, but one animal from each group (43/12 and 73/12) was positive up to 9 dpc. Viral RNA in OP fluid and retropharyngeal tonsils declined (Ct 30–39.9) from 4 to 11 dpc. The two control animals had viral RNA detected between 2 and 11 dpc in both OP fluid and retropharyngeal tonsil swabs. Viremia was detected from 2 to 4 dpc with low Ct values of viral RNA (Ct 30–39.9). The two control animals had both viral RNA detected and virus recovered from heparinized blood at 2–4 dpc.

### 3.5. Variation in Response among Individuals to Antigenic Sites on the VP2 Protein Responsible for Antigenic Drift

The outer-capsid protein VP2 of SAT2 viruses tolerates mutations in two critical and surfaced-exposed regions, i.e., residues 71–72 and 133–134. However, the biological significance of these mutations in affording protection in FMD-vaccinated animals is not yet known [31]. To investigate whether the two adjuvanted SAT2 vaccines may induce a difference in the antibody response, we measured the reactivity of the 162 dpv sera against two epitope-replaced SAT2 viruses. In the epitope-replaced viruses, residues 71–72 and 133–134 of the VP2 protein of vSAT2 were replaced with those of a disparate SAT2 virus, KNP/19/89, to yield vKNP^S2a^SAT2 and vKNP^S2b^SAT2 [31], respectively.

**Figure 6:**
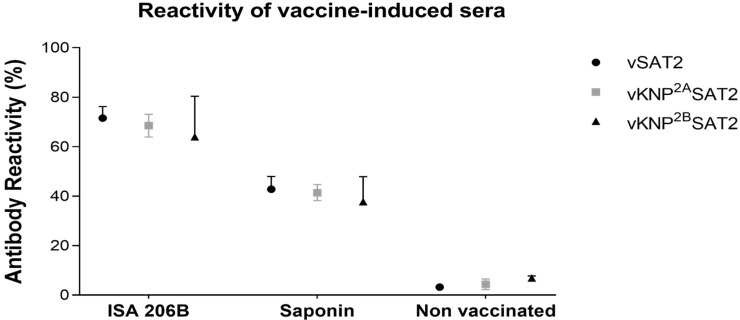
Summary of reactivity for sera collected at 162 dpv from Nguni cattle immunized twice with 2 mL of 6–8 µg dose of vSAT2-S93H BEI-inactivated, SDG-purified stabilized SAT2 antigens formulated with either Montanide ISA 206B or Quil-A Saponin adjuvant. Mean total antibody reactivity (%) measured by cELISA against antigenic sites located on the outer-capsid protein VP2 of SAT2 epitope-modified viruses. Error bars represent the standard deviation.

The binding assay revealed that all sera from SAT2-Montanide ISA 206B and SAT2-Quil-A Saponin vaccinated animals had antibodies that reacted similar to vSAT2 and the epitope-replaced viruses (Figure 6). Generally, the antibody reactivity of sera from SAT2-Montanide ISA 206B vaccinated animal sera were higher (73.6%, 69.8% and 59.8%) compared to SAT2-Quil-A Saponin vaccinated sera (44.2%, 41.8% and 39.9%) against vSAT2, vKNP^S2a^SAT2 and vKNP^S2b^SAT2 viruses. Taken together, animals vaccinated with either Montanide ISA 206B or Quil-A Saponin formulated SAT2 vaccines produced antibodies against antigenically drifted viruses with mutations in surface-exposed residues of VP2.

## 4. Discussion

FMD vaccines are notorious for eliciting a short-lived immunity in cattle in FMD-endemic regions and require re-vaccination at regular intervals of 4–6 months to ensure protective levels of antibodies [3,44]. Modern adjuvants are used to enhance the effectiveness of veterinary important vaccines. In adjuvant comparison studies, Montanide ISA 206B oil emulsified FMD vaccines elicited long-lived immunity [18,19,45] and the efficacy of a vaccine was enhanced by the addition of Saponin [26,46]. In the present study, we compared and evaluated the efficacy of two adjuvant formulations in combination with a structurally stabilized SAT2 vaccine antigen designed to have improved thermostability. The ability of a double oil emulsion (W/O/W) of Montanide ISA 206B and a Quil-A Saponin adjuvanted SAT2 vaccine to elicit protective immune responses in Nguni cattle were investigated. In the industry, it is a general practice to add AL with Saponin adjuvant for vaccine formulation [22]. However, some studies alluded to the fact that the physical structure of a vaccine antigen disintegrates following adsorption of the AL [18,28]. In this study, a semi-purified Quil-A adjuvant was used for vaccine formulation instead of the toxic crude Saponin. Therefore, AL was not added to the Quil-A adjuvant in order to protect the structural integrity and thermostability of the stabilized antigen vaccine.

The induction of neutralizing antibodies is usually considered as the most important factor to confer protective immunity against FMDV [47,48,49]. Thus, for an adjuvant to be effective, it should be able to generate neutralizing antibody responses faster, at higher levels and for a longer duration. The benefits of using oil-formulated vaccines have been well established, including the ability to induce protective immune responses as early as 7 dpv and induce long-lived antibody responses for at least six months after vaccination [3,18,19,20,22,25]. The Montanide ISA 206B adjuvanted vaccine was more effective in eliciting an anti-SAT2/ZIM/7/83 neutralizing antibody response compared to the Quil-A Saponin adjuvanted vaccine. The neutralizing antibodies in SAT2-Montanide ISA 206B vaccinated cattle were present at least 154 dpv and for 112 dpv in SAT2-Quil-A Saponin vaccinated cattle. Although no statistical difference was observed in neutralizing antibody titres after 84 dpv, the SAT2-Montanide ISA 206B vaccinated animals generally had higher total antibody levels and for longer compared to the SAT2-Quil-A Saponin vaccinated.

Subsequent to the intra-dermolingual challenge with homologous virus at 162 dpv, a strong correlation was observed between neutralizing antibody responses and clinical protection. Protection mediated by the Montanide ISA 206B adjuvanted vaccine was complete (*n* = 7), as determined by the absence of generalized lesions. However, the Quil-A Saponin adjuvanted vaccine only caused partial protection (*n* = 6). One of the cattle developed clinical signs and generalized lesions 7–11 dpc, albeit less severe than the control animals. For the protected animals in both groups, protection was not sterile and virus and viral RNA could be detected in the OP fluid and retropharyngeal tonsils 2–9 dpc. A low level (Ct > 30) of viral RNA could also be detected circulating in the blood. These results provided proof that a vaccine dose of 6–8 µg of BEI-inactivated SAT2 antigen administered twice, six weeks apart, elicits a protective immune response in cattle for at least 5 months as advised by [9].

In addition to total and neutralizing antibody titres, we measured the IgG subclasses as an in vitro marker of protection [38]. We found no significant differences between the IgG1:IgG2 ratio for the two vaccinated groups. Both vaccinated groups induced higher titres of anti-FMDV IgG1 isotype antibodies compared to IgG2 antibody responses, and IgG1 titres in the SAT2-Montanide ISA 206B vaccinated group stayed high for a longer period of time. After challenge, the kinetics of IgG1 and IgG2 isotype titres increased from 4 to 7 dpc. Capozzo et al. (1997) [38] reported that the ability of a FMD vaccine to raise a stronger IgG1 response correlates with the ability of vaccinated animals to be protected. Our data confirms evidence obtained by Scott et al. (2017b) [35] supporting the role of the IgG1 isotype in recovery and protection of cattle from FMD.

The importance of a cell-mediated immune response against FMDV infection has been reported previously [35,39,50,51]. Consequently, the role of regulating the cell-mediated T-lymphocytes was measured by IFN-γ, which is mainly produced by activated cells in anamnestic responses. IFN-γ levels measured from whole blood are extremely variable [52]; however, validation and implementation of the test was based on previous findings [39] and performed as described previously [35]. Significantly (*p* < 0.05) higher levels of systemic IFN-γ responses were elicited 14 dpv and peaked at 28 dpv in animals vaccinated with the Montanide ISA 206B adjuvanted vaccine (8.9 ± 7.4 ng/mL), followed by SAT2-Quil-A Saponin (6.6 ± 5.6 ng/mL) vaccinated animals. Systemic IFN-γ anamnestic T-cell responses were higher at 84 dpv for SAT2-Montanide ISA 206B vaccinated animals, even when total antibody responses had declined. After challenge, the recall of significantly (*p* < 0.05) higher levels of IFN-γ induced by SAT2-Montanide ISA 206B (5.8 ± 5.6 ng/mL) compared to the SAT2-Quil-A Saponin (2.1 ± 5.6 ng/mL) group 9 dpc, was observed. The unprotected animal from the SAT2-Quil-A Saponin group had a high neutralizing antibody titre (>2.1 log_10_) and IgG1 but low systemic IFN-γ response at 9 dpc. It is reasonable to suggest that the presence of a cell-mediated immunity component contributed to the extent of protection. These results demonstrate that the use of structurally stabilized SAT2 antigen with Montanide ISA 206B adjuvant for vaccine formulation has the ability to induce greater IFN-γ levels. Our data confirms the importance of IFN-γ T-cell responses in vaccine-mediated protection [39,53,54]. Moreover, the findings also showed that the quantity of IFN-γ produced post-vaccination correlates with protection against clinical FMD in vaccinated animals. Montanide ISA 206B may be a better activator of the cellular immune response and for the production of memory cells during vaccination, which were recalled following challenge of animals and thereby conferred protection in vivo.

Antigenic variation of the virus is the consequence of the high mutation rate of the virus followed by selection due to immune response evasion. The antigenic changes contribute to decreased vaccine efficacy and success of vaccination programs in the field [3,55]. We measured the reactivity of the 162 dpv sera against two epitope-replaced SAT2 viruses, vKNP^S2a^SAT2 and vKNP^S2b^SAT2, that may simulate immune escape. No significant difference in reactivity of sera from SAT2-Montanide ISA 206B or SAT2-Quil-A Saponin vaccinated animals against vSAT2, vKNP^S2a^SAT2 or vKNP^S2b^SAT2 viruses were detected. The results indicated that both vaccine-induced antibody responses were able to efficiently cross-react and neutralize viruses with amino acid changes in structurally exposed-loops in the capsid.

## 5. Conclusions

In conclusion, we have shown the efficacy of structurally stabilized SAT2 vaccine antigen emulsified with two adjuvant formulations. The results suggest that Montanide ISA 206B is superior to Quil-A Saponin adjuvant for eliciting a protective immune response in Nguni cattle with higher and longer duration of neutralizing antibodies, IgG1 isotype and systemic IFN-γ responses after one vaccination. The study also highlighted the importance of giving a primary course of SAT2 vaccines, six weeks apart, to establish a satisfactory level of immunity, followed by re-vaccination every six months.

## Data Availability

The data presented in this study are available on request from the corresponding author. The raw data were generated at the Agricultural Research Council and are not publicly available due to Institutional regulations.
